# Immunotherapy efficacy predictive tool for lung adenocarcinoma based on neural network

**DOI:** 10.3389/fimmu.2023.1141408

**Published:** 2023-03-28

**Authors:** Wei Li, Siyun Fu, Xiang Gao, Zhendong Lu, Renjing Jin, Na Qin, Xinyong Zhang, Yuhua Wu, Weiying Li, Jinghui Wang

**Affiliations:** ^1^ Cancer Research Center, Beijing Chest Hospital, Capital Medical University/Beijing Tuberculosis and Thoracic Tumor Research Institute, Beijing, China; ^2^ Department of Medical Oncology, Beijing Tuberculosis and Thoracic Tumor Research Institute/Beijing Chest Hospital, Capital Medical University, Beijing, China

**Keywords:** immunotherapy, lung adenocarcinoma, neural network, deep learning, predictive model

## Abstract

**Background:**

Remarkably, the anti-cancer efficacy of immunotherapy in lung adenocarcinoma (LUAD) has been demonstrated. However, predicting the beneficiaries of this expensive treatment is still a challenge.

**Materials and methods:**

A group of patients (N = 250) diagnosed with LUAD and receiving immunotherapy were retrospectively studied. They were randomly divided into a training dataset (80%) and a test dataset (20%). The training dataset was utilized to train neural network models to predict patients’ objective response rate (ORR), disease control rate (DCR), responders (progression-free survival time > 6 months), and overall survival (OS) possibility, which were validated by both the training and test datasets and packaged into a tool later.

**Results:**

In the training dataset, the tool scored 0.9016 area under the receiver operating characteristic (AUC) curve on ORR judgment, 0.8570 on DCR, and 0.8395 on responder prediction. In the test dataset, the tool scored 0.8173 AUC on ORR, 0.8244 on DCR, and 0.8214 on responder determination. As for OS prediction, the tool scored 0.6627 AUC in the training dataset and 0.6357 in the test dataset.

**Conclusions:**

This immunotherapy efficacy predictive tool for LUAD patients based on neural networks could predict their ORR, DCR, and responder well.

## Highlights

Remarkably, the anti-cancer efficacy of immunotherapy in lung adenocarcinoma has been demonstrated, but predicting the beneficiaries of this expensive treatment is still a challenge.

We developed a clinician-friendly tool to predict lung adenocarcinoma immunotherapy efficacy by only using patients’ demographic features and routine testing to act as predictive variables, without any additional or expensive examination.

This tool might provide some reference for clinicians on the management of lung adenocarcinoma patients’ therapy.

## 1 Introduction

Lung cancer remains one of the leading causes of cancer-related death worldwide with a 5-year relative survival rate of 23.6%, accounting for approximately one in five (18.0%) deaths (1, 2). Lung adenocarcinoma (LUAD), the most prevalent histological type of lung cancer, comprised virtually 40% of all cases, and the incidence has risen over the last few decades with progressive screening, examinations, and diagnosis (3, 4). Nearly half of LUAD patients harbor activating oncogenes, and identification of oncogenic alteration is routine in clinical practice. Targeted therapy-based precise genotyping has significantly prolonged the survival of LUAD patients with driver gene mutations and profoundly led tumor treatment strategies to a revolutionary era, which emerged and developed rapidly in the past decade (5, 6). However, a majority of patients received an initial response but eventually developed resistance to targeted therapy (6). These cases did not change much until immunotherapy appeared. In addition, patients without driver oncogenes had a poor outcome because of lacking targeted therapy when compared to those with oncogenes, although the combination of chemotherapy with bevacizumab was an optional first-line treatment.

Therapeutic advances in novel immunotherapies or immunotherapy combinations based on the interaction between the human immune system and cancer have emerged rapidly in the past few years. Remarkably, the anti-cancer efficacy and safety of immunotherapy have been demonstrated in numerous ongoing clinical trials (7–11). Among these various types of immunotherapies with different molecular targets, therapeutically targeting immune inhibitory checkpoints through the blockade of programmed cell death 1 (PD-1) or programmed cell death ligand 1 (PD-L1) has unprecedentedly led to durable responses across a broad range of human cancers, which was then widely used in the clinical practice of various solid tumors (12). PD-L1 expression detected by immunohistochemistry (IHC) is widely considered the gold standard in predicting the response for anti-PD1/PDL1 in immunotherapy (13). However, considering the complex and highly regulated nature of the immune system, only a minority of patients experienced durable benefits from these therapies. The role of PD-L1 in predicting efficacy and identifying beneficiary patients is still imperfect. To overcome this dilemma, there has been a growing interest in exploring interior biomarkers or formulating methods or tools based on external clinicopathological characteristics to predict the response of immune checkpoint inhibitors (ICIs) for discovering individualized treatment strategies tailored to patient-specific characteristics to maximize efficacy and achieve personalized medicine.

In order to address this clinical need, 250 LUAD patients with anti-PD1/PDL1 immunotherapy were retrospectively studied, and a predictive tool was developed then based on their real clinical features using a state-of-the-art neural network algorithm. The tool has been uploaded, which might provide some reference for clinicians on the management of LUAD patients.

## 2 Materials and methods

### 2.1 Data sources and study design

**Figure 1:**
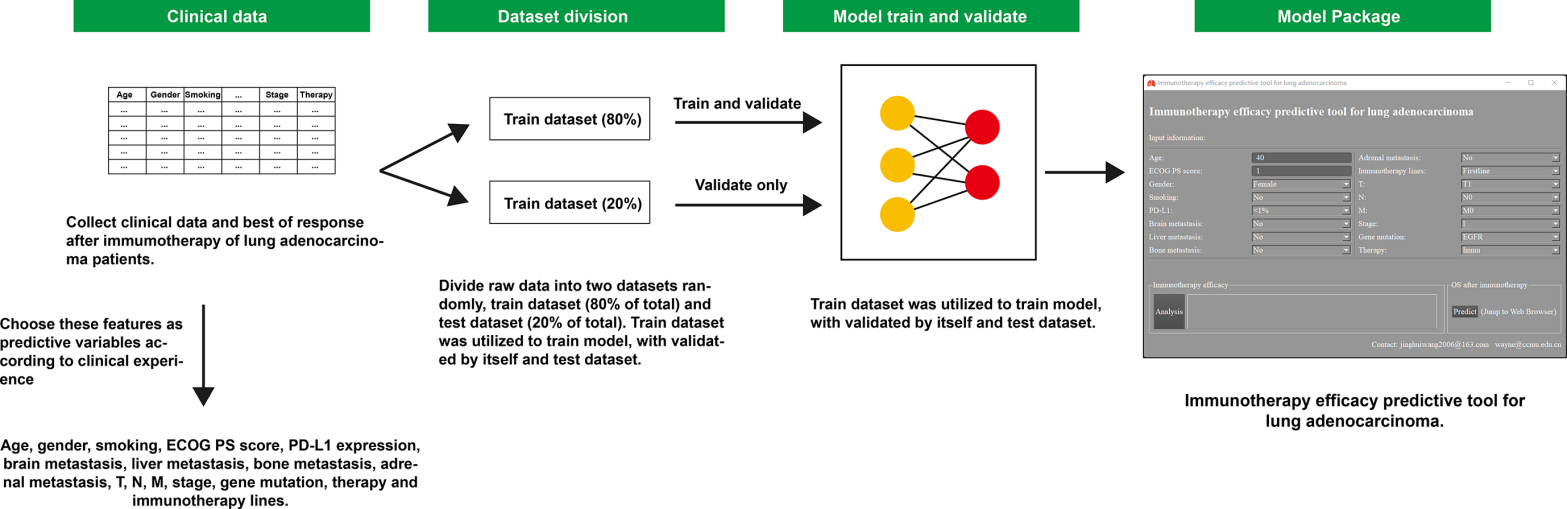
Workflow diagram of this study. ECOG, Eastern Cooperative Oncology Group; PS score, performance status score; PD-L1, programmed cell death ligand 1; OS, overall survival.

This research was designed as a retrospective cohort study. Patients diagnosed with LUAD pathologically and received immunotherapy from January 2016 to November 2021 in Beijing Chest Hospital affiliated with Capital Medical University were included. Inclusion criteria were as follows: patients had at least one evaluable lesion, receiving at least two cycles of immunotherapy, and had a response evaluation according to Response Evaluation Criteria in Solid Tumors (RECIST) v1.1. Meanwhile, patients with active autoimmune disease, symptomatic interstitial lung disease, and multiple primary pulmonary carcinomas were excluded. A total of 250 patients met the above criteria and were enrolled finally and were then followed up until lost to follow-up or death. The last follow-up time was 30 April 2022. All data were divided into two groups randomly: the training dataset (80% of the total) and test dataset (20% of the total). The training dataset was utilized to train the model, validated by itself and the test dataset (**Figure 1**). This study has been approved by the Ethics Committee of Beijing Chest Hospital affiliated with Capital Medical University.

### 2.2 Clinical features and predictive variables

According to clinical experience, we chose and collected these clinical features as predictive variables: age, gender, smoking, Eastern Cooperative Oncology Group (ECOG) performance status score (PS), PD-L1 expression, brain metastasis, liver metastasis, bone metastasis, adrenal metastasis, T, N, M, stage, gene mutation, therapy methods, and immunotherapy lines. TNM stage was evaluated in terms of the 8th American Joint Committee on Cancer (AJCC) stage. Gene mutation was classified into the following categories: EGFR, KRAS, other mutation (TP53), uncommon mutation (ALK, ROS1, RET, MET, BRAF, and HER2), and negative. Therapy methods included immunotherapy (Immu) only, immunotherapy with antiangiogenic (Antiangio), immunotherapy with chemotherapy (Chemo), or a combination of the above three treatments. Immunotherapy medicine was divided into two types: first line or subsequent lines.

There were some missing values (**Supplement Figure 1**) in certain clinical features, so we used Multivariate Imputation by Chained Equations method to impute them, with the help of R package mice (14).

### 2.3 Study outcome

The primary outcome was the objective response rate (ORR). According to patients’ best of response (BOR) to immunotherapy, they were assessed as complete response (CR), partial response (PR), stable disease (SD), or progressive disease (PD). Patients showing CR or PR were regarded as ORR. The subordinate outcome was disease control rate (DCR), responder identification, and overall survival (OS). DCR included CR, PR, and SD. Patients whose progression-free survival (PFS) time was more than 6 months after immunotherapy were considered responders. The measurement above complied with RECIST v1.1. The PFS was from the first day of immunotherapy to the date of disease progression or any cause of death. The OS was defined from the first day of immunotherapy to the date of death due to any cause.

### 2.4 Model training

Before training, numerical variables were standardized, which meant numerical variables subtracted their means and divided by their standard deviations, while categorical variables were converted into dummy variables, such as replacing gender with two dummy variables (female = 0 or male = 1). Age and ECOG PS were standardized, and the other clinical features were transformed into dummy variables.

Given this classification task, we used dense neural networks (DNNs) to predict the outcomes. ORR, DCR, and responder identification were analyzed by three DNNs independently. OS was predicted by a neural network survival model based on Katzman’s DeepSuvr theory (15). Usually, the neural network performed well in classification tasks, but it is not its forte to handle time-dependent data.

To obtain better training effectiveness, we used batch training, dropout layers, and early stopping function during the above process. Batch training meant that models were trained with several samples per training epoch. Dropout layers randomly set input units to 0 at each step during training to prevent overfitting. Early stopping function could end up training immediately as the model’s performance did not get promoted after selected training epochs. These procedures were completed in python 3.9 (https://www.python.org/).

### 2.5 Model evaluation and packaged into a tool

The receiver operating characteristic (ROC) curve and the area under the receiver operating characteristic curve (AUC) were applied to evaluate the models’ performance. The closer the ROC curve is to the upper left corner of the graph, the higher the accuracy of the model. Similarly, the closer the AUC is to 1, the better the performance of the model. A model with AUC ≥ 0.8 is considered acceptable and performed well (16). The training dataset was used to conduct models, which were then validated by both the training dataset and test dataset.

Finally, all models were packaged into an immunotherapy efficacy predictive tool for lung adenocarcinoma. This tool was a Windows 11 64-bit executable program and free for doctors and researchers to use.

### 2.6 Statistical analysis

We conducted Cox proportional hazards regression to explore the prognostic risk factors and protective factors on OS in advanced LUAD patients (III–IV stage) after immunotherapy. All statistical analyses were completed with R software (https://www.r-project.org/). Categorical features were represented by numbers and percentages, compared by chi-square or Fisher’s exact test. Skewed distribution data were represented by the median and interquartile range (IQR) and analyzed by the Wilcoxon test. A two-sided p < 0.05 was considered to be statistically significant.

## 3 Results

### 3.1 Clinical features of patients

A total of 250 patients were enrolled. They were classified into two groups—responders (41.6%, 104/250) and non-responders (58.4%, 146/250)—according to the duration of PFS. The PD-L1 expression of responders (PFS > 6 months) was 1%–49% or ≥50% mostly, while non-responders (PFS ≤ 6 months) mainly expressed PD-L1 less than 1%. Responders had less adrenal metastasis and seemed to receive first-line immunotherapy medicine more. All responders showed PR or SD without any PD, but non-responders tended to achieve SD or PD the most. Responders had a longer OS than non-responders. In brief, responders tended to achieve a higher PD-L1 expression and less adrenal metastasis and used first-line immunotherapy medicine more commonly. Otherwise, responders and non-responders had similar features in other aspects, like age, gender, smoking, ECOG PS score, brain metastasis, liver metastasis, bone metastasis, T, N, M, stage, mutation, and therapy (**Table 1**).

**Table 1:** Clinical features of patients.

	>6 months	≤6 months	Test method	p-Value
	(N = 104)	(N = 146)		
	N (%)			
Age			Wilcoxon	0.9703
Median (IQR)	62 (56, 67.25)	63 (56, 68)		
Gender			Chi-square	0.0629
Female	23 (22.12)	48 (32.88)		
Male	81 (77.88)	98 (67.12)		
Smoking			Chi-square	0.6300
No	41 (39.42)	62 (42.47)		
Yes	63 (60.58)	84 (57.53)		
ECOG PS score			Wilcoxon	0.4343
Median (IQR)	1 (1, 1)	1 (1, 1)		
PD-L1			Chi-square	0.0077**
<1%	19 (18.27)	51 (34.93)		
1%–49%	31 (29.81)	29 (19.86)		
≥50%	31 (29.81)	33 (22.6)		
Unknown	23 (22.12)	33 (22.6)		
Brain metastasis			Chi-square	0.1056
No	89 (85.58)	113 (77.4)		
Yes	15 (14.42)	33 (22.6)		
Liver metastasis			Chi-square	0.1377
No	96 (92.31)	126 (86.3)		
Yes	8 (7.69)	20 (13.7)		
Bone metastasis			Chi-square	0.7906
No	73 (70.19)	105 (71.92)		
Yes	30 (28.85)	40 (27.4)		
Unknown	1 (0.96)	1 (0.68)		
Adrenal metastasis			Chi-square	0.0326*
No	94 (90.38)	120 (82.19)		
Yes	8 (7.69)	25 (17.12)		
Unknown	2 (1.92)	1 (0.68)		
T			Fisher’s exact	0.3132
1	5 (4.81)	5 (3.42)		
1a	1 (0.96)	1 (0.68)		
1b	2 (1.92)	5 (3.42)		
1c	6 (5.77)	3 (2.05)		
2	14 (13.46)	24 (16.44)		
2a	3 (2.88)	9 (6.16)		
2b	8 (7.69)	4 (2.74)		
3	15 (14.42)	15 (10.27)		
4	48 (46.15)	75 (51.37)		
Unknown	2 (1.92)	5 (3.42)		
N			Chi-square	0.0502
0	20 (19.23)	17 (11.64)		
1	8 (7.69)	7 (4.79)		
2	47 (45.19)	57 (39.04)		
3	24 (23.08)	55 (37.67)		
Unknown	5 (4.81)	10 (6.85)		
M			Chi-square	0.1745
0	24 (23.08)	21 (14.38)		
1	4 (3.85)	6 (4.11)		
1a	22 (21.15)	34 (23.29)		
1b	18 (17.31)	18 (12.33)		
1c	31 (29.81)	62 (42.47)		
Unknown	5 (4.81)	5 (3.42)		
Stage			Fisher’s exact	0.5256
I	1 (0.96)	1 (0.68)		
II	0 (0)	2 (1.37)		
III	23 (22.12)	24 (16.44)		
IV	79 (75.96)	118 (80.82)		
Unknown	1 (0.96)	1 (0.68)		
Mutation			Chi-square	0.2061
EGFR	20 (19.23)	31 (21.23)		
KRAS	29 (27.88)	28 (19.18)		
Other	9 (8.65)	7 (4.79)		
Uncommon mutation	8 (7.69)	14 (9.59)		
Negative	28 (26.92)	55 (37.67)		
Unknown	10 (9.62)	11 (7.53)		
Therapy			Chi-square	0.5195
Immu	17 (16.35)	32 (21.92)		
Immu + Antiangio	9 (8.65)	12 (8.22)		
Immu + Chemo	56 (53.85)	80 (54.79)		
Immu + Chemo + Antiangio	22 (21.15)	22 (15.07)		
Immunotherapy lines			Chi-square	0.0466*
Firstline	61 (58.65)	67 (45.89)		
Subsequent lines	43 (41.35)	79 (54.11)		
BOR			Chi-square	<0.001***
PR	58 (55.77)	34 (23.29)		
SD	46 (44.23)	66 (45.21)		
PD	0 (0)	46 (31.51)		
PFS			Wilcoxon	<0.001***
Median (IQR)	9.96 (7.56, 14.64)	3.07 (1.62, 4.51)		
OS			Wilcoxon	<0.001***
Median (IQR)	15.65 (11.97, 20.82)	8.41 (5.55, 12.47)		

Only non-missing values were statistically analyzed.

PFS, progression-free survival; IQR, interquartile range; ECOG PS score, Eastern Cooperative Oncology Group performance status score; PD-L1, programmed cell death ligand 1; Another gene mutation, TP53; Uncommon gene mutation, ALK, ROS1, RET, MET, BRAF, and HER2; Immu, immunotherapy; Antiangio, antiangiogenic therapy; Chemo, chemotherapy; BOR, best of response; PR, partial response; SD, stable disease; PD, progressive disease; OS, overall survival.

*p < 0.05; **p < 0.01; ***p < 0.001.

**Figure 2:**
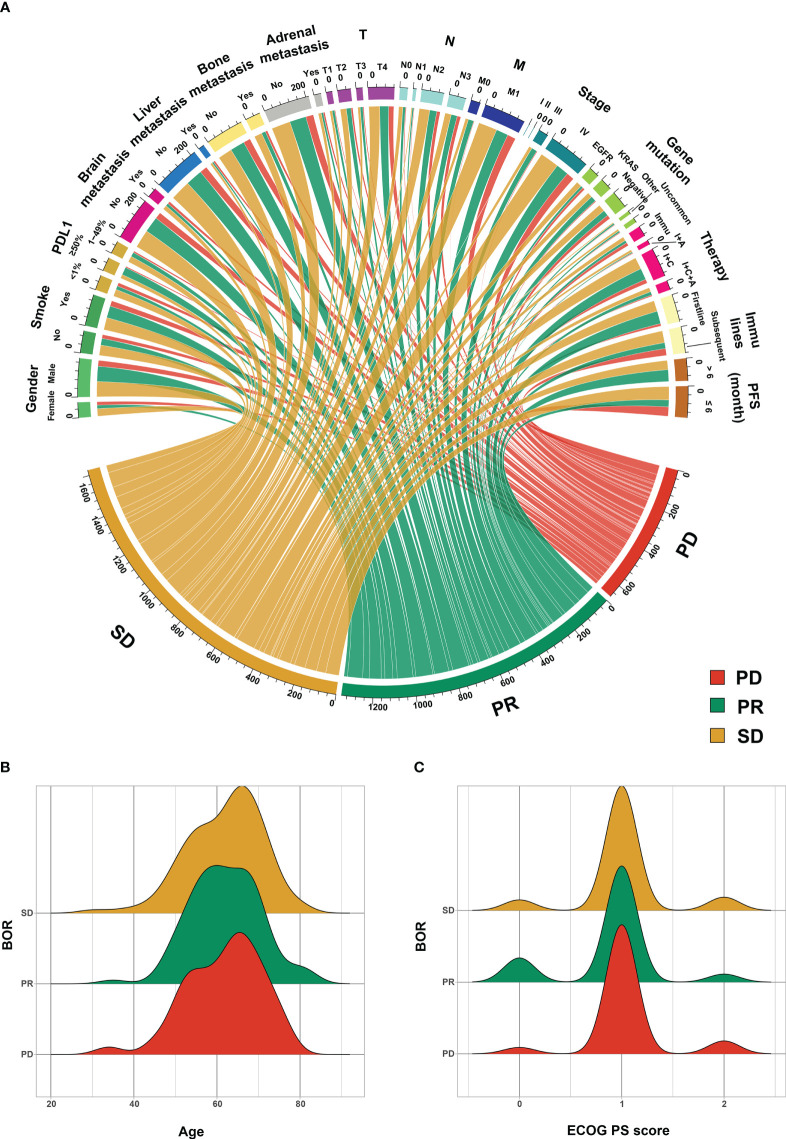
The source of progressive disease (PD) partial response (PR) and stable disease (SD) patients. **(A)** categorical features, **(B)** age, and **(C)** Eastern Cooperative Oncology Group (ECOG) performance status score (PS score). PD-L1, programmed cell death ligand 1; Other gene mutation, TP53; Uncommon gene mutation, ALK, ROS1, RET, MET, BRAF, and HER2; Immu, immunotherapy, I+A, immunotherapy and antiangiogenic therapy; I+C, immunotherapy and chemotherapy therapy; I+C+A, immunotherapy, chemotherapy, and antiangiogenic therapy; PFS, progression-free survival; BOR, best of response.

**Figure 2** visually illustrates the source of PR, SD, and PD patients. **Figure 2A** shows the relation between categorical features and BOR, while **Figures 2B, C** display the numerical features’ distribution (age and ECOG PS score) in different BOR groups.

### 3.2 Model performance

**Figure 3:**
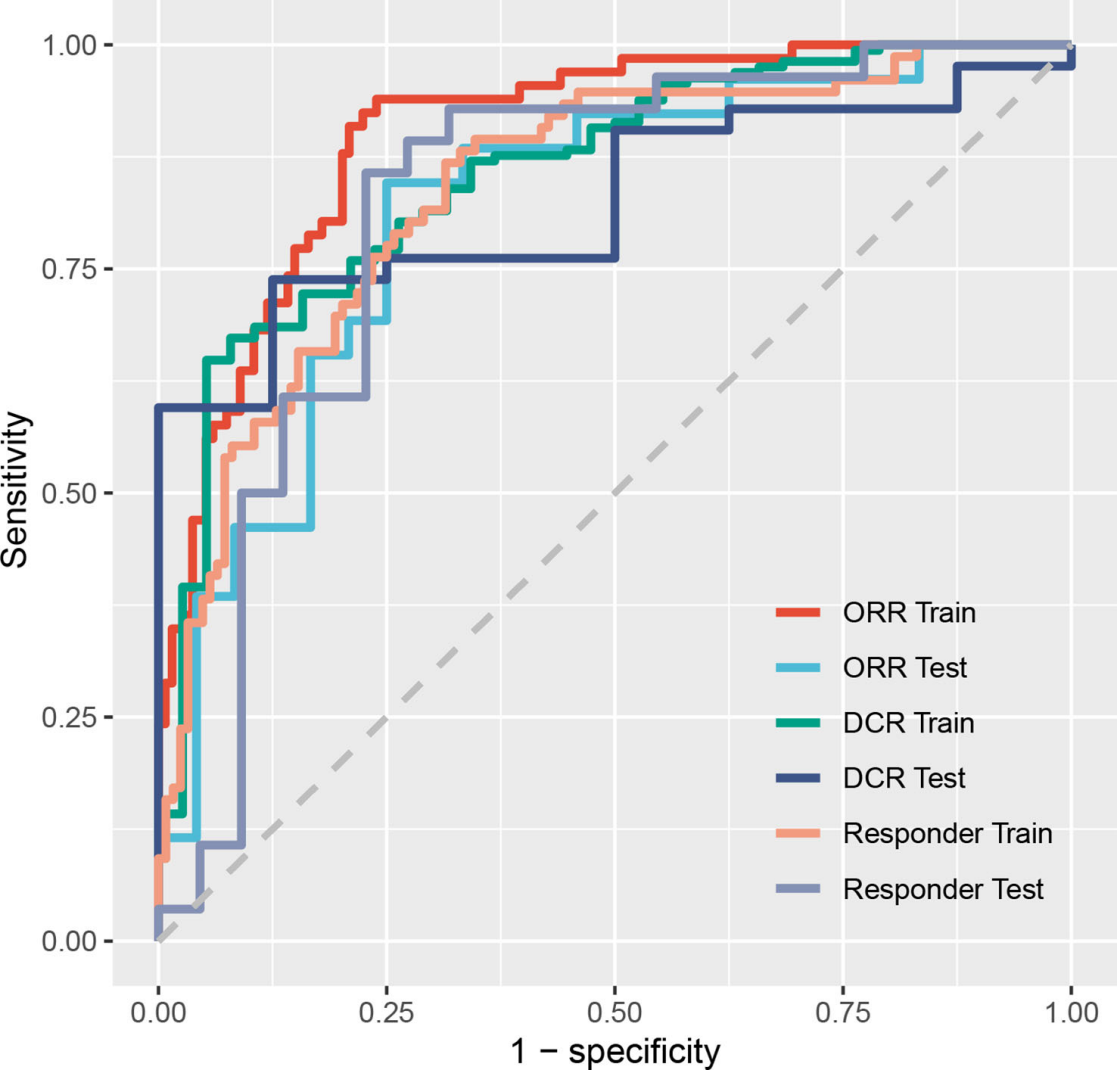
Receiver operating characteristic curves of models on predicting ORR, DCR, and responder. ORR, objective response rate; DCR, disease control rate; Responder, progression-free survival time was more than 6 months.

The immunotherapy efficacy predictive models showed satisfactory performance. In the training dataset, models scored 0.9016 (95% confidence interval (CI): 0.8592–0.9441) on ORR judgment, 0.8570 (95% CI, 0.7923–0.9218) on DCR, and 0.8395 (95% CI, 0.7829–0.8960) on responder prediction. In the test dataset, the models scored 0.8173 (95% CI, 0.6959–0.9388) on ORR, 0.8244 (95% CI, 0.7000–0.9488) on DCR, and 0.8214 (95% CI, 0.6903–0.9526) on responder determination (**Table 2** and **Figure 3**). As for OS prediction, model performance was very ordinary, scoring 0.6627 (95% CI, 0.6613–0.6640) in the training dataset and 0.6357 (95% CI, 0.6331–0.6384) in the test dataset (**Table 2**).

**Table 2:** The performance of immunotherapy efficacy predictive tool.

	Train dataset		Test dataset	
	AUC	95% CI	AUC	95% CI
ORR	0.9016	0.8592–0.9441	0.8173	0.6959–0.9388
DCR	0.8570	0.7923–0.9218	0.8244	0.7000–0.9488
Responder	0.8395	0.7829–0.8960	0.8214	0.6903–0.9526
OS	0.6627	0.6613–0.6640	0.6357	0.6331–0.6384

AUC, area under receiver operating characteristic curve; CI, confidence interval; ORR, objective response rate; DCR, disease control rate; Responder, progression-free survival time is more than 6 months; OS, overall survival.

### 3.3 Predictive tool

**Figure 4:**
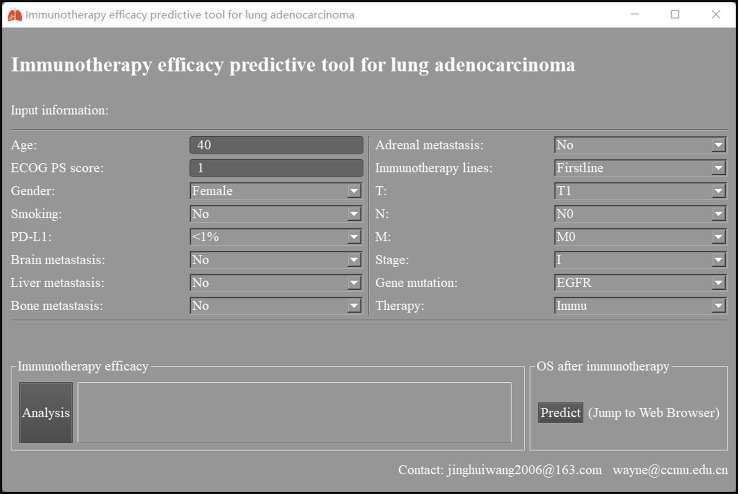
The interface of immunotherapy efficacy predictive tool for lung adenocarcinoma based on neural network. ECOG PS score, Eastern Cooperative Oncology Group performance status score; PD-L1, programmed cell death ligand 1; Immu, immunotherapy; OS, overall survival.

Immunotherapy efficacy predictive models for LUAD based on the neural network were packaged into a predictive tool. As **Figure 4** shows, after related clinical information is inputted, models will calculate and report this patient’s possibility of ORR, DCR, and responder. The tool can also predict this patient’s OS after immunotherapy when the Predict button is clicked.

### 3.4 Survival analysis

In our clinical practice, the majority of LUAD patients receiving immunotherapy were primarily advanced ones (stage III–IV). Therefore, advanced LUAD patients’ data were utilized to orient risk and protective factors on their OS after missing values were omitted. As **Supplement Figure 2** illustrates, patients with bone metastasis had shorter survival, while patients with PR and SD had better prognoses than those with PD.

## 4 Discussion

In recent years, numerous clinical trials have strongly supported immunotherapy for routine clinical use (7–11). According to KEYNOTE-024, pembrolizumab has shown a durable, long-term survival benefit with a 5-year OS rate of 31.9%, further providing meaningful evidence of immunotherapy’s clinical application (17). Currently, whether monotherapy or combination with chemotherapy plus antiangiogenic therapy or not, immunotherapy has been commonly used in clinical practice as the first-line or subsequent treatment for LUAD patients without targetable mutations.

With the widely applied and remarkable achievement of immunotherapy in LUAD, there is an increasingly great concern for how to accurately select the best candidates (10, 18). The current standards for selection still have certain limitations. First, although PD-L1 expression in tumor tissue has been advocated as a standard for anti-PD1/PD-L1 immunotherapy and moderate concordance has been shown by previous studies in PD-L1 assay results, the results are still influenced by multiple factors such as intra-tumoral heterogeneity of PD-L1 expression, cellularity, and more three-dimensional cell clusters in cytology samples (19). Second, researchers were committed to discovering new biomarkers, such as tumor mutational burden (TMB). TMB, which is defined as the total number of non-synonymous somatic mutations in tumor cells, has been found to be related to tumor antigenicity, and might be an independent biomarker of immunotherapy outcome (20). Patients with high TMB and receiving immunotherapy were significantly correlated with longer PFS and higher ORR, compared to those receiving traditional chemotherapy (21, 22). Based on KEYNOTE-158 (NCT02628067), TMB was approved as a reference index for the treatment of solid tumors using pembrolizumab (23). However, TMB has not been widely endorsed because of its inability to predict OS and its inconsistent predictive efficacy in all cancer types (21, 24). Furthermore, the accuracy of TMB evaluation could be affected by different methods of specimen handling (25). Third, researchers began to use more sophisticated and better algorithms to handle medical issues, like predicting immunotherapy efficacy. The combination of chemotherapy and immunotherapy is more popular because of a better outcome, but still, there is a lack of predictive markers. Predictive tools or models based on artificial intelligence have been increasingly emerging in recent years. T. Araujo and colleagues used a convolutional neural network to diagnose breast cancer based on hematoxylin and eosin-stained breast biopsy images, and the model achieved 83.3% accuracy and 95.6% sensitivity (26). C. Du and colleagues applied a neural network to classify imbalanced electrocardiosignal data, achieving 98.45% accuracy and 97.03% sensitivity (27). T. Dratsch developed and validated a neural network to identify the 30 most common categories of plain radiographs, and the model showed 90.3% overall accuracy (28). Deep learning is gradually recognized by clinicians. Therefore, we chose a neural network to predict LUAD patients’ immunotherapy efficacy.

In this study, we aimed to develop a predictive tool to help identify the ideal candidates for immunotherapy in LUAD, which was packaged into a Windows file later and convenient to use. We collected the clinical features of 250 patients included, dividing them into the training dataset and the test dataset randomly. Then, we utilized the training dataset to conduct models and validated them with both the training and test datasets. The predictive performance of our tool is satisfactory, with AUC > 0.8 on most evaluated items (ORR, DCR, and responder possibility), except OS. Notably, our model scored 0.9016 and 0.8173 AUC in ORR classification, 0.8570 and 0.8244 in DCR prediction, and 0.8395 and 0.8214 in responder identification. These showed that our tool had good efficacy in predicting LUAD patients’ immunotherapy benefits.

More and more researchers have devoted themselves to developing a model to predict the benefit of immunotherapy for cancer patients. Some investigators focused on building a model on the database of gene mutation. Jie Peng et al. developed a predictive model associated with the durable clinical benefit of ICIs to LUAD patients, using deep neural networks based on somatic mutations tested by whole-exome sequencing and targeted next-generation sequencing (29). The model exhibited 0.884 AUC in the training set and 0.914 AUC in the two validation sets. Regretfully, the authors did not make the neural network available as a clinician-friendly website or software. Meanwhile, other researchers were dedicated to the information on the tumor microenvironment. Jinteng Feng et al. constructed a nomogram associated with CD8^+^ T cells to predict survival rates and immunotherapy benefits of stage III LUAD patients (30). A moderate performance showed in an online database that their prediction model had approximately 0.649–0.709 AUC, but a survival difference between the two groups (high- and low-risk groups) was not observed (30). A similar study used the next-generation sequencing method mainly; these predictive models might be limited to being widely applied for lack of gene sequencing data, which might be too expensive for most patients. Compared to those studies, our study provided novel insights into predicting the clinical benefit of ICIs for LUAD patients. We only used patients’ demographic features and routine testing to act as predictive variables, without any additional or expensive examination. Moreover, to facilitate clinicians’ use, we have packaged the model into available Windows software.

In spite of the advantages, our tool still had some limitations. Regrettably, the efficacy of our tool to predict the patients’ OS remained unsatisfactory. Increasing the sample size may improve the predictive efficacy of our tool. Moreover, it is better to use prospective clinical data to validate our tool again.

In summary, we developed a neural network model to predict immunotherapy efficacy for LUAD patients using general clinical features and packaged it into Windows software. This tool showed satisfactory performance and has been uploaded in **Supplement File 1**, which might help optimize immunotherapy management for LUAD patients. More biomarkers may further enhance the model’s predictive accuracy.

## Data availability statement

The original contributions presented in the study are included in the article/**Supplementary Materials**. Further inquiries can be directed to the corresponding authors.

## Ethics statement

This research has been approved by the Ethics Committee of Beijing Chest Hospital affiliated with Capital Medical University.

## Author contributions

JW, WYL, WL, SF, and XG came up with the idea for the study. SF, XG, ZL, RJ, NQ, XZ, and YW collected the data and conducted the follow-up. WL carried out the statistical analysis. WL, SF, and XG wrote the manuscript. JW and WYL edited and reviewed the manuscript. All authors discussed the results and commented on the manuscript. All the authors contributed to the article and approved the submitted version.

## Supplementary material

Supplementary Figure 1 The distribution of missing values. ECOG PS score, Eastern Cooperative Oncology Group performance status score. PD-L1, programmed cell death ligand 1. BOR, best of response. PFS, progression free survival. OS, overall survival.

Supplementary Figure 2 Risk factors and protective factors on overall survival in III-IV stage lung adenocarcinoma patients after immunotherapy. PS score, performance status score. PD-L1, programmed death-ligand 1. Other mutation, TP53. Uncommon mutation, ALK, ROS1, RET, MET, BRAF, HER2. Immu, immunotherapy. Antiangio, antiangiogenic. Chemo, chemotherapy. BOR, best of response. PD, progressive disease. PR, partial response. SD, stable disease. *P<0.05, **P<0.01, ***P<0.001.

Supplement File 1 The installation program of immunotherapy efficacy predictive tool.

Supplement File 2 The raw clinical data.
