# Supplementary figures and images for: Immunotherapy efficacy predictive tool for lung adenocarcinoma based on neural network
Source: Front Immunol. 2023 Mar 28;14:1141408. doi: 10.3389/fimmu.2023.1141408 (PMC10086240; doi:10.3389/fimmu.2023.1141408)

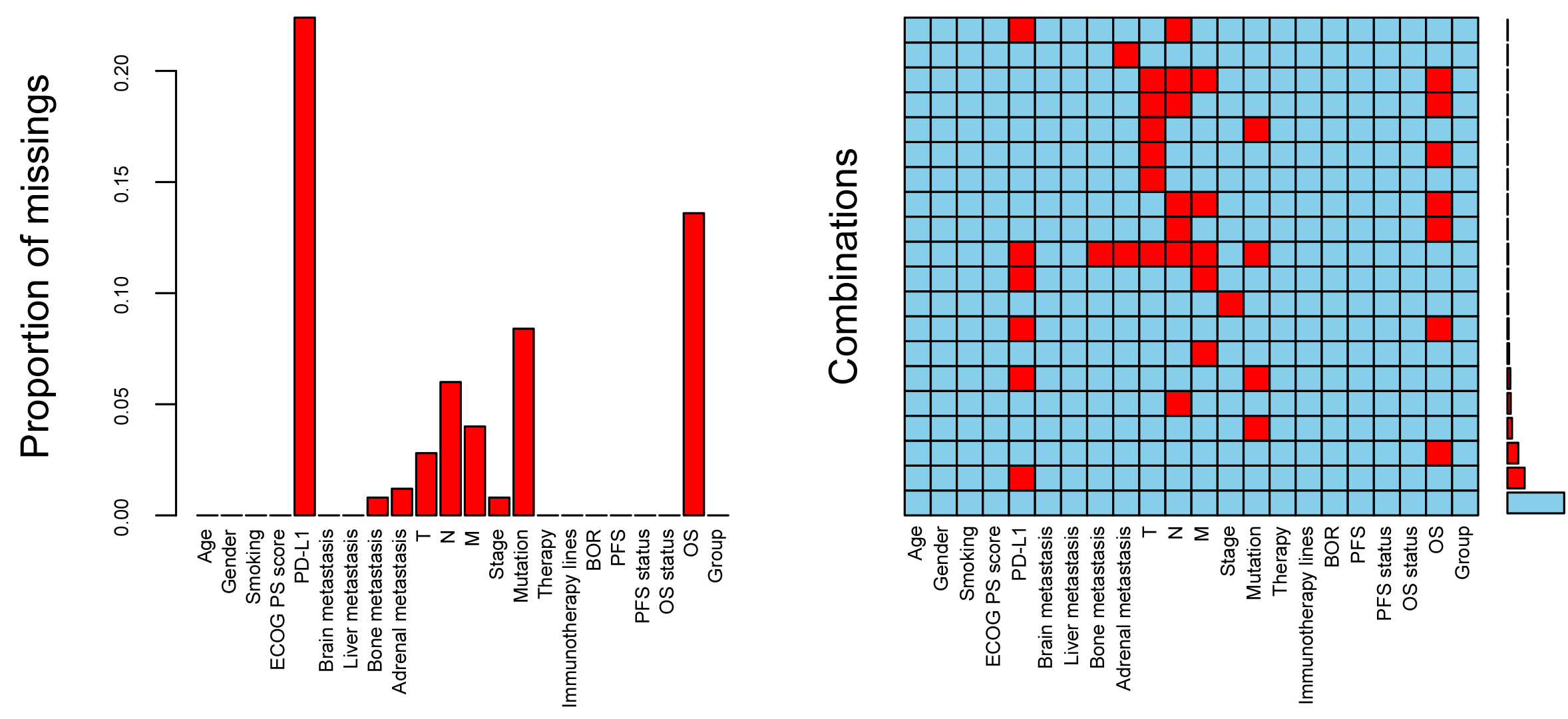

Supplement: Supplementary Figure 1 — The distribution of missing values. ECOG PS score, Eastern Cooperative Oncology Group performance status score. PD-L1, programmed cell death ligand 1. BOR, best of response. PFS, progression free survival. OS, overall survival. [file Image_1.tif]

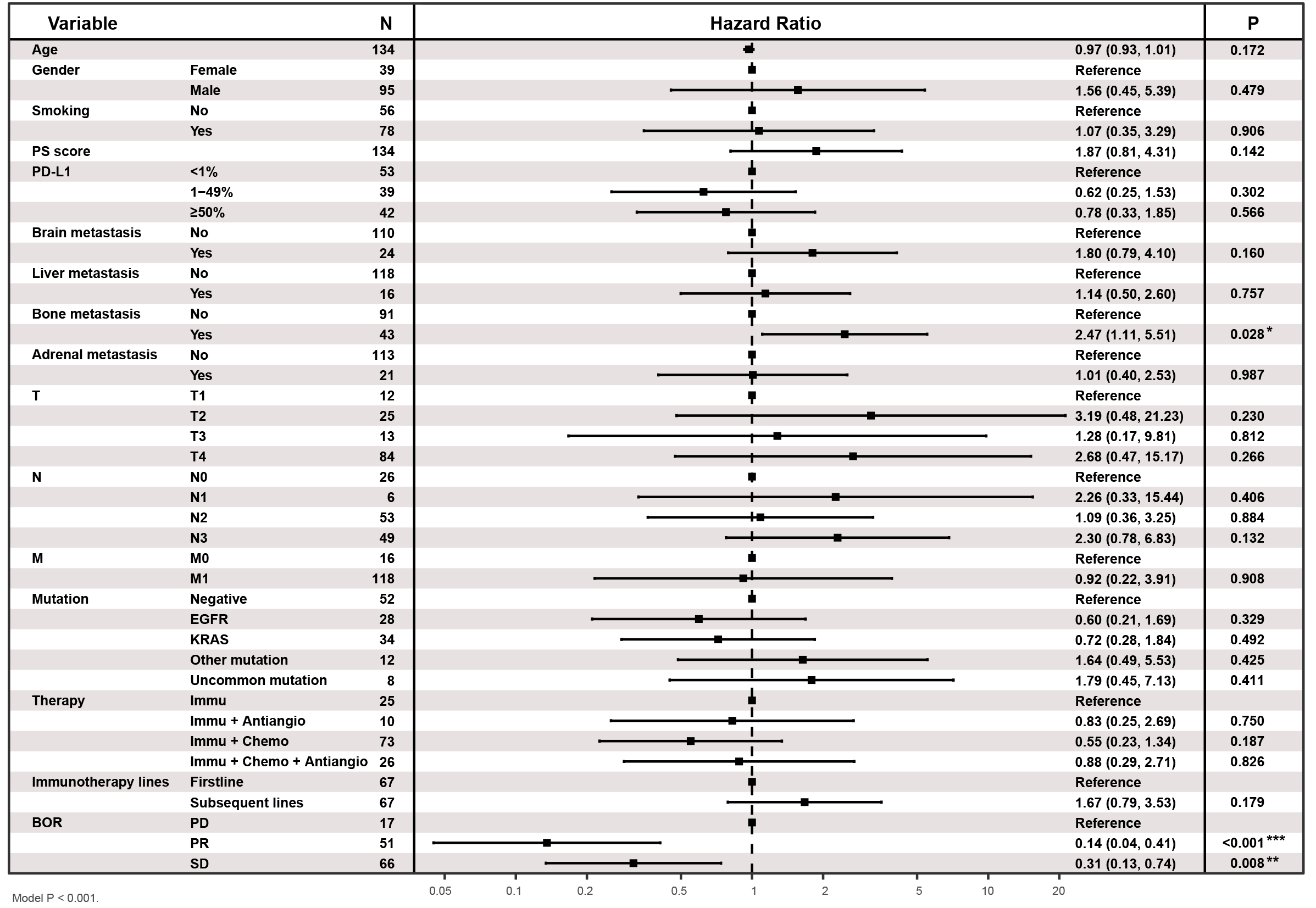

Supplement: Supplementary Figure 2 — Risk factors and protective factors on overall survival in III-IV stage lung adenocarcinoma patients after immunotherapy. PS score, performance status score. PD-L1, programmed death-ligand 1. Other mutation, TP53. Uncommon mutation, ALK, ROS1, RET, MET, BRAF, HER2. Immu, immunotherapy. Antiangio, antiangiogenic. Chemo, chemotherapy. BOR, best of response. PD, progressive disease. PR, partial response. SD, stable disease. *P<0.05, **P<0.01, ***P<0.001. [file Image_2.tif]
